# A Transient Left Atrial Appendage Filling Defect During Anticoagulation Interruption After Ischemic Stroke with Progressive Hemorrhagic Transformation: A Case Report

**DOI:** 10.3390/reports9030314

**Published:** 2026-09-17

**Authors:** Woochan Choi, Yang-Ha Hwang

**Affiliations:** Department of Neurology, School of Medicine, Kyungpook National University, Daegu 41944, Republic of Korea

**Keywords:** atrial fibrillation, hemorrhagic transformation, left atrial appendage, anticoagulation interruption

## Abstract

**Background and Clinical Significance:** Anticoagulation timing after atrial fibrillation (AF)-related ischemic stroke is uncertain when hemorrhagic transformation (HT) is progressive. Although early direct oral anticoagulant (DOAC) initiation is supported in selected patients, evidence is limited for parenchymal hemorrhage or progressive HT. **Case Presentation:** A 72-year-old man receiving aspirin therapy, in whom AF was newly diagnosed on admission, presented with multi-territorial ischemic stroke and HT. He was febrile, with a C-reactive protein level of 15.54 mg/dL, and underwent serial abdominal computed tomography (CT) to investigate the source of fever. Given concern regarding intracranial bleeding associated with HT, apixaban 2.5 mg twice daily was initiated on day 4 but discontinued on day 6 because of progressive HT. No antithrombotic therapy was subsequently administered. CT on day 2 showed no left atrial appendage (LAA) filling defect. On day 10, after reduction in inflammatory activity, repeat CT demonstrated a new 15-mm LAA filling defect radiologically suggestive of thrombus. The defect was no longer visible on day 16. Transesophageal echocardiography on day 19 showed no intracardiac thrombus. Apixaban was resumed on day 35 after radiographic HT resolution. He received antithrombotic therapy for only 2 of the first 35 days after stroke onset. **Conclusions:** A transient LAA filling defect was observed during a prolonged antithrombotic-free interval and was not visualized on follow-up CT. Although contemporaneous dedicated cardiac imaging was unavailable and slow flow or contrast-mixing artifact could not be excluded, this serial observation raises the possibility of a transient thrombotic phenomenon during anticoagulation interruption.

## 1. Introduction and Clinical Significance

Atrial fibrillation (AF) is a major cause of cardioembolic ischemic stroke. The risk of recurrent embolism is highest in the early period after stroke, while the risk of hemorrhagic transformation (HT) is also greatest during this interval, particularly after large or multi-territorial infarction. Anticoagulation is the principal preventive treatment for recurrent embolism, but it may worsen intracranial bleeding in patients with unstable HT.

Randomized trials have supported early direct oral anticoagulant (DOAC) initiation after AF-related ischemic stroke in selected patients [1,2,3,4]. However, evidence is substantially less secure in patients with parenchymal hemorrhage. In ELAN, parenchymal and remote hemorrhage were protocol-defined exclusion criteria; however, the 56 of 1933 participants (2.8%) with these findings were identified only on subsequent central imaging review and had therefore been enrolled despite the protocol. In this small subgroup, early treatment was associated with a higher risk of poor functional outcome at 90 days (adjusted odds ratio, 2.92; 95% confidence interval, 1.01–8.91), despite no symptomatic intracranial hemorrhage. The investigators proposed subclinical HT progression as a speculative explanation; given the subgroup size, this association should be regarded as hypothesis-generating rather than causal [5].

Patients in whom HT is present before anticoagulant exposure and progresses after treatment were not specifically represented in these trials, and evidence directly applicable to this clinical scenario is limited. In clinical practice, anticoagulation may be withheld while radiographic stability of HT is reassessed. In some patients, the duration of interruption may be influenced by serial imaging, uncertainty regarding hemorrhage stability, availability of cardiac testing, and transitions between healthcare settings. We report a patient with progressive HT in whom a left atrial appendage (LAA) filling defect was absent on baseline CT, appeared during an extended antithrombotic-free interval, and was no longer visible on follow-up CT. The finding was radiologically suggestive of a possible transient thrombotic phenomenon, although its nature could not be definitively established.

## 2. Case Presentation

A 72-year-old man with hypertension, diabetes mellitus, dyslipidemia, and myocardial infarction treated with coronary stenting 11 years previously presented with sudden visual disturbance and dizziness. He had been receiving aspirin 100 mg/day before admission. Neurologic examination showed right homonymous hemianopia and mild dysarthria. The National Institutes of Health Stroke Scale score at presentation was 5. Atrial fibrillation was newly diagnosed on admission and was paroxysmal; sinus rhythm was documented on electrocardiography later during the hospital course. The CHA_2_DS_2_-VASc score was 4 (hypertension, diabetes mellitus, vascular disease, and age 65–74 years). Brain magnetic resonance imaging on day 1 showed acute infarction in the left posterior cerebral artery territory involving the parieto-occipital area, with confluent petechial hemorrhagic transformation without mass effect (ECASS HI2) [6]. A concurrent acute infarction was also identified in the right superior cerebellar artery territory. Aspirin was discontinued on admission because of the HT. The patient weighed 74 kg, and his serum creatinine concentration was 1.4 mg/dL, with estimated creatinine clearance of approximately 50 mL/min.

**Figure 1:**
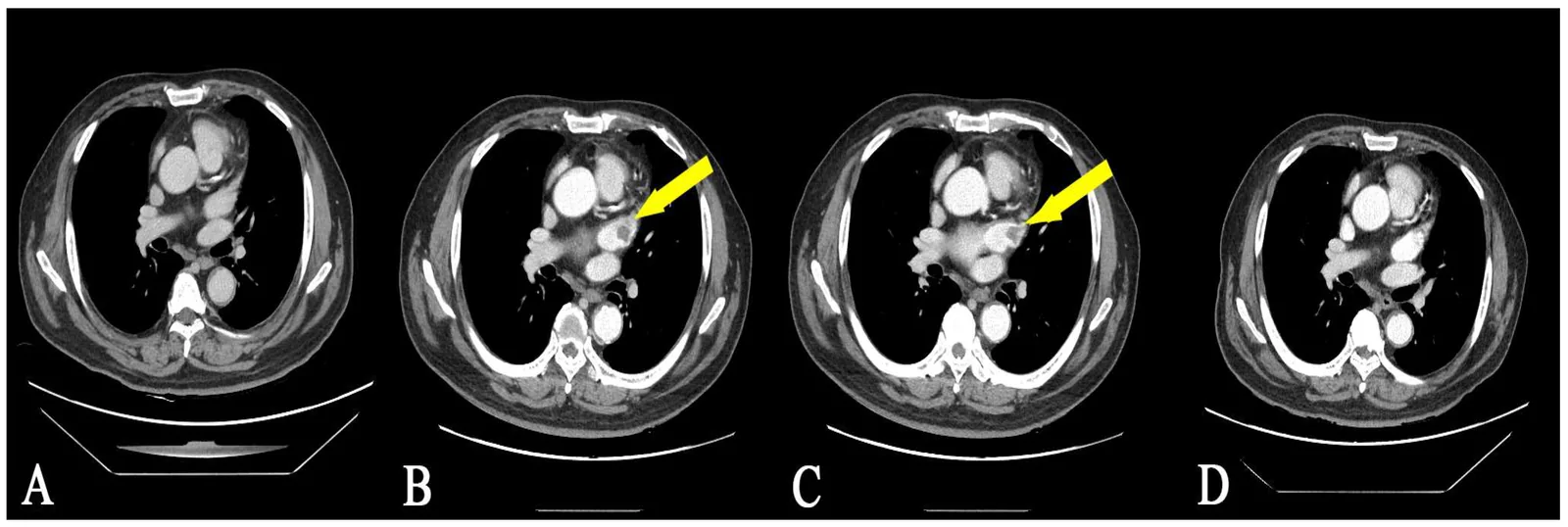
Serial contrast-enhanced computed tomography images of the left atrial appendage obtained from abdominal CT examinations. (**A**) Day 2. No filling defect is identified in the left atrial appendage on CT. (**B**,**C**) Day 10. A 15-mm filling defect is visible in the left atrial appendage (arrows), reported as possible embolic material and radiologically suggestive of thrombus. (**D**) Day 16. The previously identified filling defect is no longer visible.

He was febrile at admission, with a C-reactive protein (CRP) concentration of 15.54 mg/dL and a procalcitonin concentration of 0.165 ng/mL. Contrast-enhanced abdominal CT on day 2, performed to investigate the source of fever, revealed multiple hypoattenuating lesions in both kidneys and the spleen. The findings suggested pyelonephritis with possible renal abscesses; renal and splenic infarctions remained alternative considerations. Ceftriaxone was initiated. The LAA was included in the CT scan range, and no filling defect was observed (Figure 1A). Transthoracic echocardiography on day 4 showed a dilated left atrium without other apparent structural cardiac abnormality, although assessment was limited by arrhythmia and a poor acoustic window.

Follow-up brain imaging showed slight regression of the HT, and apixaban was initiated on day 4. Given concern regarding intracranial bleeding associated with HT, apixaban was initiated at an individualized reduced dose of 2.5 mg twice daily. The patient met none of the labeled dose-reduction criteria for apixaban, which recommend 2.5 mg twice daily only when at least two of the following are present: age ≥ 80 years, body weight ≤ 60 kg, or serum creatinine ≥ 1.5 mg/dL. This was therefore an individualized off-label dose reduction. Follow-up brain CT on day 6 showed progression of HT to PH1 within the left parieto-occipital infarct, observed after initiation of apixaban, prompting discontinuation of the drug.

Blood cultures obtained on five occasions were negative, and procalcitonin remained low. Despite antibiotic treatment, the renal and splenic lesions remained indeterminate. CRP decreased to 0.68 mg/dL by day 11. Repeat abdominal CT on day 10, performed because of persistent abdominal symptoms, demonstrated a new 15-mm LAA filling defect, reported as possible embolic material and radiologically suggestive of thrombus (Figure 1B,C). Anticoagulation was not resumed because of the recent progression of HT.

Follow-up chest and abdominal CT on day 16 showed that the LAA filling defect was no longer visible (Figure 1D). The renal and splenic lesions were unchanged or slightly decreased in size; evaluation of enhancement was limited on all three examinations, and both acute pyelonephritis with abscess formation and renal infarction remained possible. C-reactive protein rose modestly to 3.19 mg/dL on day 19, whereas procalcitonin remained low at 0.083 ng/mL. Antibiotic therapy was escalated to meropenem, and the consulting infectious disease service recommended reconsideration of non-infectious causes of the renal and splenic lesions. Transesophageal echocardiography (TEE), performed on day 19 because of scheduling limitations, showed no intracardiac thrombus or valvular vegetation. A saline contrast study was positive (grade 2/4), without directly visualized shunt flow.

**Figure 2:**
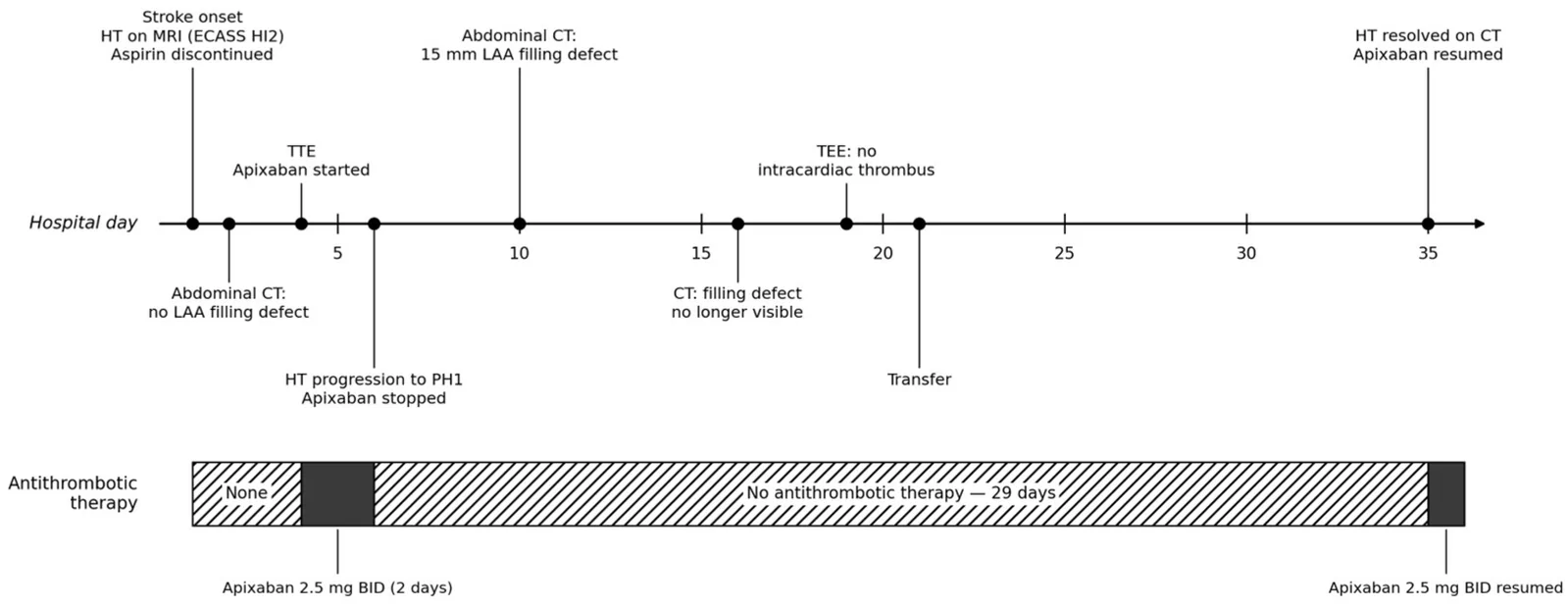
Clinical timeline over the first 35 days after stroke onset. Upper panel: key clinical and imaging events. Lower bar: antithrombotic therapy status, showing that the patient received antithrombotic therapy on only 2 of the first 35 days. ECASS, European Cooperative Acute Stroke Study; HI2, hemorrhagic infarction type 2; HT, hemorrhagic transformation; LAA, left atrial appendage; PH1, parenchymal hematoma type 1; TEE, transesophageal echocardiography; TTE, transthoracic echocardiography.

The patient was transferred to another institution on day 21 for continued treatment of suspected infection, with a modified Rankin Scale score of 2. No antithrombotic therapy was prescribed at transfer. At outpatient review on day 35, non-contrast brain CT showed resolution of HT without new ischemic lesions, and apixaban was restarted at 2.5 mg twice daily because of concern for recurrent HT. During the first 35 days after stroke onset, he therefore received apixaban for 2 days and had no antithrombotic treatment for 33 days (Figure 2). At the next outpatient visit on day 70, apixaban was increased to 5 mg twice daily. He remained on this dose until the last prescription, approximately 12 months after stroke onset, without recurrent hemorrhagic or thromboembolic events; he was subsequently lost to follow-up.

## 3. Discussion

This case may have captured a process that is ordinarily invisible in routine practice. An LAA filling defect was absent on day 2, appeared on day 10 during an antithrombotic-free interval, and was no longer visible on day 16. The patient had no recurrent clinical stroke or recognized systemic embolism. Without repeated abdominal CT that fortuitously included the heart, both the appearance and disappearance of this abnormality would have remained unknown.

The transient LAA filling defect could not be confirmed as thrombus because it was detected on contrast-enhanced abdominal CT rather than electrocardiography-gated cardiac CT, and neither delayed-phase cardiac CT nor TEE was performed while the defect was visible [7]. Slow flow, delayed LAA opacification, and contrast-mixing artifact may mimic thrombus on early-phase CT. All three examinations were performed on the same scanner using the institution’s standard contrast-enhanced abdomen-pelvis protocol at 120 kVp with 2.5-mm slice thickness, and the left atrial appendage was within the scan range on each; however, detailed contrast injection parameters and acquisition delay could not be retrieved retrospectively, so differences in appendage opacification related to contrast timing cannot be entirely excluded. We therefore describe this finding as a transient LAA filling defect radiologically suggestive of thrombus rather than definite LAA thrombus [8].

Nevertheless, the serial imaging pattern provides a clinically relevant visual record of a transient LAA filling defect observed during a 33-day interruption of antithrombotic therapy in a patient with AF and multi-territorial infarction. Although the finding was not diagnostically definitive for thrombus, the change in its visibility across serial imaging underscores a potential limitation of routine clinical assessment: transient LAA abnormalities may not be captured when imaging is obtained only at isolated time points. The findings establish a temporal association only, and no causal relationship between interruption of anticoagulation and the appearance of the filling defect can be inferred. Within this limitation, the observation may warrant careful reassessment of thromboembolic risk when anticoagulation is interrupted, particularly in patients with AF and prior embolic events.

What the serial imaging documents is a change in the visibility of a filling defect; whether this reflected true thrombus formation and resolution or a transient contrast-mixing phenomenon cannot be determined. If it represented thrombus, this case cannot establish the frequency of such events, determine whether it resolved through endogenous fibrinolysis or subclinical embolization, or prove that interruption of anticoagulation caused it. Its contribution is therefore hypothesis-generating rather than causal: it illustrates a possible transient thrombotic phenomenon during a period without antithrombotic protection and highlights the need for prospective studies using standardized cardiac imaging. Follow-up was available for approximately 12 months after stroke onset; longer-term outcomes could not be assessed. Follow-up brain imaging was limited to non-contrast CT, so small recurrent infarcts may not have been detected.

Infection and embolism remained competing explanations for the renal and splenic lesions, and the distinction was never resolved: antibiotic therapy was escalated on day 19, while the consulting infectious disease service simultaneously advised that non-infectious causes be reconsidered. Blood cultures were repeatedly negative, procalcitonin remained low, and TEE showed no vegetation. Moreover, the LAA filling defect appeared when CRP had fallen from 15.54 mg/dL at admission to 0.68 mg/dL by day 11. This temporal pattern does not favor active systemic inflammation as the principal explanation for the filling defect, although preceding infection or inflammation may have contributed to a prothrombotic state. These renal and splenic lesions therefore cannot be used as evidence supporting either an infectious or an embolic mechanism.

The clinical course raises two management considerations. First, progressive HT after initiation of anticoagulation places such patients outside the population in whom early DOAC initiation has been well studied. During the subsequent 33-day interruption of antithrombotic therapy, thromboembolic risk is likely to have persisted. Second, apixaban was initiated and later resumed at 2.5 mg twice daily as individualized clinical decisions made in response to concern about recurrent HT. The patient met none of the labeled dose-reduction criteria at either time point; this therefore represents an off-label, individualized dose reduction rather than a guideline-based decision. The subsequent increase to 5 mg twice daily at outpatient follow-up was likewise an individualized clinical decision based on demonstrated clinical and radiographic stability, rather than a validated dosing strategy. This case provides no evidence supporting the use of reduced-dose apixaban outside the established dose-reduction criteria after HT. Rather, it illustrates the uncertainty surrounding both the timing and the dose of anticoagulation when therapy is resumed after progressive HT.

## 4. Conclusions

A transient LAA filling defect, radiologically suggestive of thrombus, was observed during an extended antithrombotic-free interval in a patient with AF-related multi-territorial ischemic stroke and progressive HT. The finding was detected on non-gated abdominal CT, and contemporaneous dedicated cardiac imaging was unavailable; slow flow and contrast-mixing artifact could not be excluded. This hypothesis-generating observation raises the possibility of a transient thrombotic phenomenon but does not establish thrombus formation, causality, or an optimal strategy for anticoagulation timing or dose. Its value lies in the serial imaging record itself, which captured the temporal evolution of a finding that would not have been apparent from imaging at any single time point. During periods in which anticoagulation is withheld for progressive HT, thromboembolic risk is likely to persist and may warrant careful clinical reassessment.

## Data Availability

All relevant data are included within the article. Additional de-identified information can be made available from the corresponding author upon reasonable request, in accordance with patient confidentiality requirements.

## Abbreviations

The following abbreviations are used in this manuscript:

AF	Atrial fibrillation
CT	Computed tomography
CRP	C-reactive protein
DOAC	Direct oral anticoagulant
HT	Hemorrhagic transformation
LAA	Left atrial appendage
TEE	Transesophageal echocardiography
